# Indirect Effects of Ploidy Suggest X Chromosome Dose, Not the X:A Ratio, Signals Sex in *Drosophila*

**DOI:** 10.1371/journal.pbio.0050332

**Published:** 2007-12-27

**Authors:** James W Erickson, Jerome J Quintero

**Affiliations:** Department of Biology, Texas A&M University, College Station, Texas, United States of America; Adolf Butenandt Institute, Germany

## Abstract

In the textbook view, the ratio of X chromosomes to autosome sets, X:A, is the primary signal specifying sexual fate in *Drosophila*. An alternative idea is that X chromosome number signals sex through the direct actions of several X-encoded signal element (XSE) proteins. In this alternative, the influence of autosome dose on X chromosome counting is largely indirect. Haploids (1X;1A), which possess the male number of X chromosomes but the female X:A of 1.0, and triploid intersexes (XX;AAA), which possess a female dose of two X chromosomes and the ambiguous X:A ratio of 0.67, represent critical tests of these hypotheses. To directly address the effects of ploidy in primary sex determination, we compared the responses of the signal target, the female-specific *SxlPe* promoter of the switch gene *Sex-lethal*, in haploid, diploid, and triploid embryos. We found that haploids activate *SxlPe* because an extra precellular nuclear division elevates total X chromosome numbers and XSE levels beyond those in diploid males. Conversely, triploid embryos cellularize one cycle earlier than diploids, causing premature cessation of *SxlPe* expression. This prevents XX;AAA embryos from fully engaging the autoregulatory mechanism that maintains subsequent *Sxl* expression, causing them to develop as sexual mosaics. We conclude that the X:A ratio predicts sexual fate, but does not actively specify it. Instead, the instructive X chromosome signal is more appropriately seen as collective XSE dose in the early embryo. Our findings reiterate that correlations between X:A ratios and cell fates in other organisms need not implicate the value of the ratio as an active signal.

## Author Summary

In the fruit fly, *Drosophila*, chromosomal signals determine sex. Diploid flies with two X chromosomes are female, whereas those with one X are male. Conventionally, it is thought that the ratio of the number of X chromosomes to autosomes (X:A) constitutes the signal, because triploid flies bearing two X chromosomes and three sets of autosomes (XX;AAA) are intersexual. Under this model, the X:A signal is defined as the balance between a set of X-linked “numerator” proteins that promote female development and autosomally encoded “denominator” proteins that counteract the numerator elements. Although the X:A signal is a textbook standard, only one strong denominator element exists, and it cannot account for the effects of altered chromosome number (ploidy) on sex. To understand how X and autosome doses influence sex, we examined haploids (1X;1A) and triploids during the brief embryonic period when sex is determined. We found that ploidy affects sex indirectly by increasing in haploids, or decreasing in triploids, the number of embryonic cell cycles in which chromosomal sex is assessed. Our findings indicate that the fly sex-determination signal is more accurately viewed as a function of the number of X chromosomes rather than as a value of the X:A ratio.

## Abstract 3

Sex is determined by X chromosome number rather than by the value of the X:autosome ratio in *Drosophila*.

## Introduction

Animals distinguish between numbers or kinds of sex chromosomes both to determine sex and to compensate for unequal gene expression between heterogametic (XY and ZW) and homogametic (XX and ZZ) sexes. In *Drosophila* and Caenorhabditis elegans, sex and dosage compensation are linked through genetic pathways that exploit transient differences in the expression of several dose-dependent X-linked genes to lock in developmentally stable regulatory states (reviewed in [1]). In mammals, sex is determined by the presence or absence of the Y chromosome, but X chromosome dosage compensation is initiated after a quantitative assessment of X chromosome dose (see [2]). It is thought, in all these cases, that X number is assessed in conjunction with overall ploidy, because changes in the number of autosomal sets relative to X chromosomes affects sexual development or dosage compensation.

The link between autosome dose and sex determination in *Drosophila* was established in the 1920s when Calvin Bridges showed that triploid flies bearing two X chromosomes and three sets of autosomes (XX;AAA) develop as sexual mosaics [3,4]. This led to the concept that the somatic sex-determination signal is not simply X dose, but rather, the ratio between the number of X chromosomes and the sets of autosomes in the zygote, the X:A ratio. Accordingly, in flies, an X:A of 0.5 (XY;AA) is said to signal male, and an X:A of 1.0 (XX;AA) to signal female, whereas the intermediate X:A of 0.67 (XX;AAA) is an ambiguous signal that some cells interpret as male and others as female. The conventional view for the fly is that each cell in the embryo reads the value of the X:A ratio by measuring the dose of X-linked “numerator” gene products with reference to autosomally encoded “denominator” proteins to set the appropriate on or off activity state of the master sex-determination gene *Sex-lethal* (*Sxl*) (see [5–7]). When the X:A equals 1.0, the numerator proteins activate the transiently acting establishment promoter, *SxlPe*, creating a pulse of SXL, an RNA binding protein [8]. In contrast, when the X:A is 0.5, the inhibitory effect of the denominator proteins predominates so that *SxlPe* is left inactive and no early SXL is made. Once the X:A ratio has been assessed, *SxlPe* is permanently inactivated, and the maintenance promoter, *SxlPm*, is turned on in both sexes; however, only in females is SXL present to bind the *SxlPm*-derived transcripts and direct them to be spliced into functional *Sxl* mRNA. Thereafter, *Sxl* is maintained in the on state by autoregulatory RNA splicing [9,10]. In males, where no early SXL is present, transcripts from *SxlPm* are spliced by default to a nonfunctional form and SXL is never produced. Once set in the stable, autoregulated, on (female) or off (male) state, *Sxl* controls all subsequent events in somatic sexual development through control of downstream effectors of sex determination and dosage compensation (reviewed in [1,5,11–13]).

Although four X-linked genes that fulfill all the requirements of X:A numerator elements and one autosomal gene that meets the definition of a denominator element have been identified (see [1,14]), the notion that the X:A ratio is the instructive sex-determining signal relies primarily on correlations between sexual phenotypes and X:A ratios in flies with abnormal ploidy (Table 1). Given modern understanding of the molecules involved and the fact that the system evolved to determine sex in diploid animals, where autosome dose never varies, some have argued that it makes more sense to consider primary sex determination as an X chromosome–counting process, rather than as an X:A sensing one [15–18]. In this alternate view, the male or female dose of X chromosomes is defined by the collective concentrations of four X-linked signal element (XSE) proteins: SisA, Scute, Unpaired, and Runt, that function to activate *SxlPe*. Proper assessment of XSE concentration by *SxlPe* depends on numerous protein cofactors present in equal amounts in XY and XX embryos. These cofactors include an autosomal gene product, Deadpan (Dpn) [17–19], but numerous maternally supplied proteins are thought to play the predominant quantitative roles in defining the effective XSE dose. The classic finding, that XX;AAA flies are intersexual, is explained by the XSE-sensing model as the consequence of triploidy affecting proper assessment of X dose and not as implying active participation of a set of autosomal factors analogous to the XSEs.

**Table 1:**
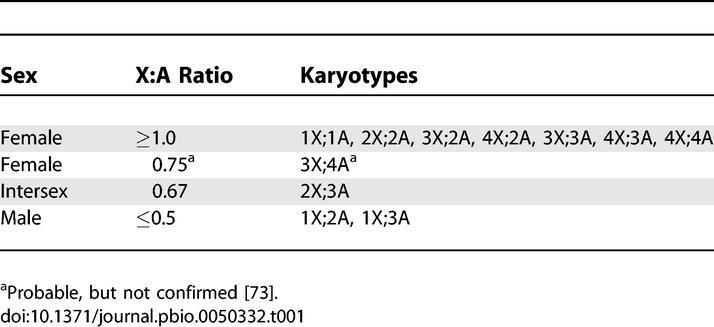
Karyotypes Examined for Sexual Phenotypes (Modified from [7])

Haploids, 1X:1A, represent the most stringent test of the X:A model because they possess the male X number, but the female X:A ratio, and develop as females [20–24]. If the XSE-sensing alternative is indeed a more accurate representation of mechanism than is the X:A model, it must explain why the X:A ratio appears to be a better predictor of ultimate sexual phenotype than is X chromosome number.

To answer the question of whether the fly determines its sex by counting X chromosomes or by reading the X:A ratio, we reexamined sex determination in haploids and triploids. Because adult sexual phenotypes need not reflect the fidelity of sex-signal assessment [1,15], we monitored the transcriptional response of the direct sex-signal target, *SxlPe*, during the early embryonic period when chromosomal sex is assessed. Our results suggest that haploids become female, not because their X:A equals one, but rather because they undergo an extra nuclear division cycle that prolongs the period in which XSE genes are expressed. Remarkably, increased ploidy affects the sexual fate of triploids in a reciprocal manner. We found that triploid embryos cellularize one cell cycle earlier than diploids. The intersexual phenotype of XX;AAA flies thus appears to be, in part, a consequence of there being too little time to accumulate a sufficient concentration of XSE proteins to strongly activate *SxlPe* in all nuclei. Our findings provide direct experimental support for the notion that XSE gene dose, and not the value of the X:A ratio, is the molecular signal that determines sex in *Drosophila*.

## Results

In diploid flies, chromosomal sex is determined during the rapid syncytial nuclear divisions that precede formation of the cellular blastoderm [8,25,26]. The establishment phase of sex determination begins about 65–75 min after fertilization, during nuclear cycles 8 and 9, with somatic transcription of the XSE genes *sisA* and *scute* [16,27,28]. This first phase continues with female-specific activation of *SxlPe* at about 105 min, during cycle 12, and ends approximately 40 min later when *SxlPe* is shut off in the first minutes of cycle 14 [8,16,29]. The maintenance phase begins immediately thereafter with activation of *SxlPm* and the transition to the stable autoregulatory mRNA splicing mode of *Sxl* expression [8,17]. Thus, sexual fate has been determined well before the completion of somatic cellularization and the onset of gastrulation.

### Haploid Development and an Alternative to the X:A Signal Model

Early development of haploids mirrors that of diploids with an important exception. Haploid embryos undergo an extra syncytial division after cycle 13 and cellularize during nuclear cycle 15 [30–32]. We wondered whether this extra division cycle might provide an explanation for the female character of haploids. In essence, we asked whether haploids become female because their X:A ratio equals one, or because the extra cycle allows more time for XSE protein accumulation and *SxlPe* activation. The two hypotheses make different predictions. If the value of the X:A ratio is determining, *SxlPe* should be expressed at the same times in X;A haploid and XX;AA diploid embryos because the X:A ratio is the same in both cases. In contrast, if the extra haploid cycle is responsible for female development, *SxlPe* activation should be delayed in haploid embryos because they have fewer X chromosomes, and thus, lower amounts of XSE products than equivalently staged diploid females.

To generate haploid embryos, we used two different X-linked recessive maternal-effect mutations: *maternal haploid* (*mh*) and *sesame* (*ssm*) [30,33]. Homozygous *mh* or *ssm* females produced eggs in which the paternal genetic contribution is lost in the earliest divisions, resulting in the development of haploid embryos [34,35] (see Materials and Methods). Sibling females heterozygous for *mh* or *ssm* produced normal embryos that served as diploid controls. We used in situ hybridization to monitor *SxlPe* activity. Key to our analysis was the ability to see focused dots of nuclear staining representing the nascent *SxlPe* transcripts on the X chromosomes, as well as the accumulated cytoplasmic *Sxl* mRNA [17,27,29,36].

### Delayed Expression from *SxlPe* in Haploids

**Figure 1:**
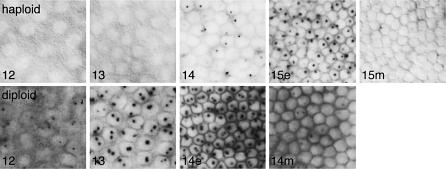
Delayed Onset of *Sxl* RNA Synthesis in Haploids Haploid 1X;1A (top row) and diploid XX;AA (bottom row) embryos were stained following in situ hybridization. Dots represent nascent transcripts from *SxlPe* in surface nuclei. Nuclear cycles (12–15) are indicated; e and m denote early (≤5 min) and mid (≤20 min) stages of the cellularization cycles. Haploid embryos cellularize during nuclear cycle 15 and diploids during cycle 14. Cycle 12 and haploid cycle 13 embryos were illuminated with visible and UV light to highlight DAPI-stained nuclei. Embryos were progeny of sibling *mh^1^/mh^1^*or *mh^1^*/FM3 females and *mh^1^*/Y males.

**Figure 2:**
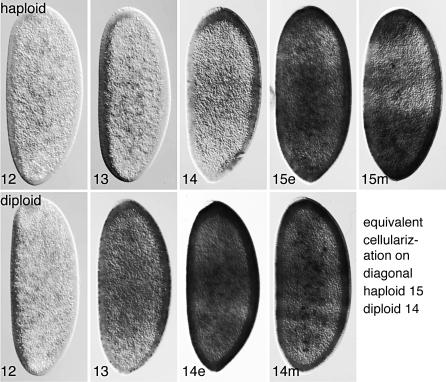
Accumulation of *SxlPe* mRNA in Precellular Embryos Haploid 1X;1A (top row) and diploid XX;AA (bottom row) embryos were stained following in situ hybridization to detect *SxlPe*-derived transcripts. Nuclear cycles 12–15 are indicated; e and m denote early (≤5 min) and mid (≤20 min) stages of cellularization cycles. Embryos were progeny of sibling *mh^1^/mh^1^*or *mh^1^*/FM3 females and *mh^1^*/Y males. Orientation is dorsal to the left and anterior to the bottom to place time on the horizontal axis.

Haploid embryos exhibited a striking delay in the onset of *SxlPe* activity as compared to diploids (Figures 1 and 2). In diploid females, *SxlPe* was first activated during nuclear cycle 12. As diploids progressed through cycle 13, the nuclear dots stained more intensely and cytoplasmic *Sxl* mRNA was first seen. Strong *Sxl* expression continued during the first few minutes of cycle 14, with maximum nuclear and cytoplasmic *Sxl* RNA staining occurring before the formation of the membrane cleavage furrows (Figures 1 and 2). In contrast, in haploid embryos, *SxlPe* activation was delayed until cycle 14. No *Sxl* transcripts were seen in haploid cycles 12 or 13, and the pattern of *Sxl* expression in haploid cycle 14 resembled that seen in diploids during the onset of transcription. In diploid females, activation of *SxlPe* is a stochastic process occurring independently on each X in each nucleus during cycle 12 [27]. Like diploid cycle 12 females, early haploid cycle 14 embryos were mosaics with respect to the proportion of expressing nuclei, consistent with our observation that *SxlPe* expression is initiated during haploid cycle 14. As haploid cycle 14 progressed, a greater proportion of nuclei expressed *SxlPe*; nonetheless, all cycle 14 haploids contained some nuclei with no detectable *Sxl* expression (Figure 1 and unpublished data).

Whereas activation of *SxlPe* was delayed in haploids, peak expression and shutoff occurred at comparable phases during the cellularization cycles of haploids (cycle 15) and diploids (cycle 14) (Figures 1 and 2). In both cases, maximum nuclear dot staining intensity and peak accumulation of *SxlPe* mRNA occurred before the formation of the membrane cleavage furrow and thereafter declined rapidly in a somewhat nonuniform pattern. Based on the similar timing of the cellularization process in haploids and diploids [32], we estimate that *SxlPe* is expressed maximally during the first 5 to 10 min of the cellularization cycles and that it is shut off in nearly all nuclei approximately 10 min later.

The process of *Sxl* activation in haploids thus appears to fit the predictions of XSE-sensing models and contradict those of the X:A signal hypothesis. If the X:A ratio were the signal, *SxlPe* would have been expressed from cycle 12 until early in the cellularization cycles of both X;A and XX;AA embryos. Instead, *SxlPe* was active in haploids only in cycles 14 and 15. This suggests that it is the extra nuclear cycle that allows haploids to become female, presumably by allowing XSE products to rise above the levels found in diploid male embryos.

### Prolonged XSE Expression in Haploids

To determine whether the XSE genes are transcribed through the extra haploid cycle, and whether haploid XSE mRNA levels eventually exceed those found in diploid males, we analyzed in detail the expression of the key XSE gene, *scute*. The *scute* locus, also known as *sisterlessB* (*sisB*), encodes a transcriptional activator that dimerizes with maternally supplied *daughterless* protein to bind to and activate *SxlPe* [37]. Quantitatively, *scute* is the most important XSE gene and is needed to activate *SxlPe* in all regions of the embryo [14,18,38,39].

**Figure 3:**
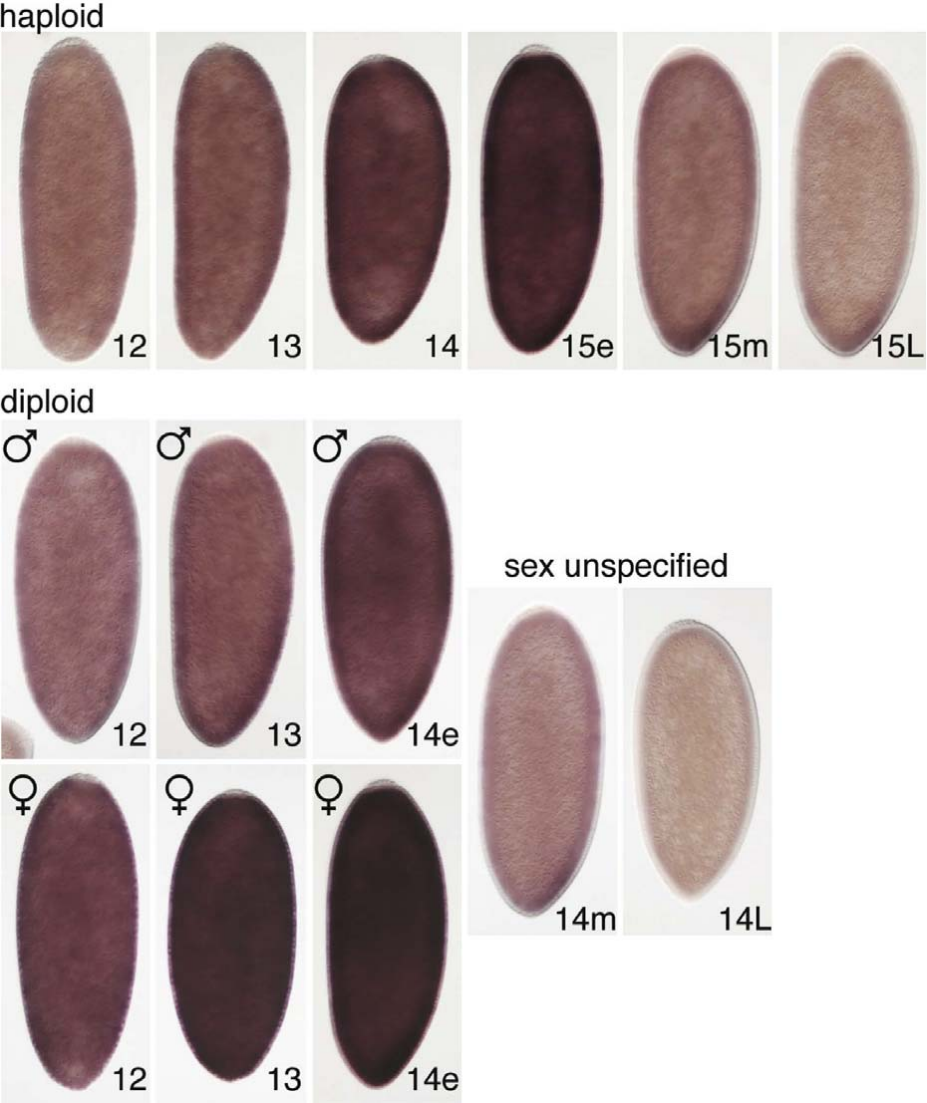
Time Course of *scute* mRNA Accumulation in Haploid and Diploid Embryos Haploid (top row) and diploid male (middle row) and female (bottom row) embryos are shown after in situ hybridization to detect *scute* mRNA. Nuclear cycles (12–15) are marked. Notations e, m, and L indicate early (≤5 min), mid (≤15 min), and late (≥30 min) times after onset of cellularization cycles. After early cycle 14, diploid male and female embryos could not be distinguished based on *scute* mRNA staining. Embryos were progeny of sibling *ssm^185b^/ssm^185b^* or *ssm^185b^*/FM3 females and *ssm^185b^*/Y males. Embryo orientation is dorsal to the left and anterior to the bottom.

Consistent with earlier findings [16,28], low-level *scute* expression could be detected at nuclear cycle 9 in both diploid and haploid embryos, but cytoplasmic *scute* mRNA was first readily apparent in cycle 11 (unpublished data). In diploids, we could reliably distinguish sex-specific differences in *scute* mRNA from cycle 12 through the first minutes of cycle 14, with female embryos expressing approximately twice the amount of *scute* mRNA as equivalently staged males (Figure 3). As cycle 14 progressed beyond the point when *SxlPe* is active, *scute* mRNA staining rapidly declined, and it was no longer possible to discriminate between male and female embryos based on *scute* mRNA levels. In combination with previous reports for *scute* mRNA [16] and protein [39], our data confirm that *scute* is expressed in direct proportion to gene dose in the precellular embryo.

In haploid embryos, *scute* mRNA levels mimicked those seen in diploid males from cycle 12 until the beginning of cycle 14. However, instead of declining immediately thereafter, *scute* mRNA levels increased throughout haploid cycle 14, reaching a peak in the first minutes of cycle 15. Importantly, at the stage when *SxlPe* was active, in haploid cycles 14 and 15, the amount of *scute* mRNA in haploids appeared to surpass the maximum levels observed in diploid males (Figure 3). Thus, the expression pattern of *scute* fits the predictions of XSE-sensing models: expression begins at the correct stage and *scute* mRNA levels increase with time and nuclear number. The maximum *scute* mRNA levels observed in haploids exceed those present in diploid males and closely match the peak levels found in diploid females during early cycle 14 (Figure 3).

### Triploid Intersexes and the Effect of Autosome Dose

We interpret the sex-determining events occurring in haploid and diploid embryos as strongly supporting the hypothesis that X chromosome dose, as defined by threshold XSE protein concentrations, is the signal that directs sexual development. Our interpretation, however, leaves unexplained the phenomenon that started the notion of the X:A ratio as the sex signal: the mosaic intersexual phenotype of XX;AAA flies [3,4]. Simply put, if X number and XSE concentrations are paramount, why are XX;AAA flies intersexes rather than females?

To experimentally address the question of how triploidy impacts the initiation of sex determination, we examined the process of *Sxl* activation in triploid embryos. To generate the triploid embryos needed, we exploited the *gynogenetic-2; gynogenetic-3* double mutant (*gyn-2; gyn-3*); so named because it can be used to produce diploid offspring with no paternal genetic contribution [40]. Gynogenetic progeny arise because *gyn-2; gyn-3* females produce a small fraction of diploid eggs that, when fertilized by nonfunctional sperm from *ms(3)K81* mutant males, develop as clones of their mothers. If these rare diploid eggs are, instead, fertilized by normal sperm, they initiate development as XXX;AAA or XXY;AAA triploids [40,41]. Experimentally, this has the advantage of generating triploid embryos without the extensive aneuploidy resulting from crosses with flies carrying compound autosomes (see [42]).

### Triploid Embryos Cellularize Prematurely

**Figure 4:**
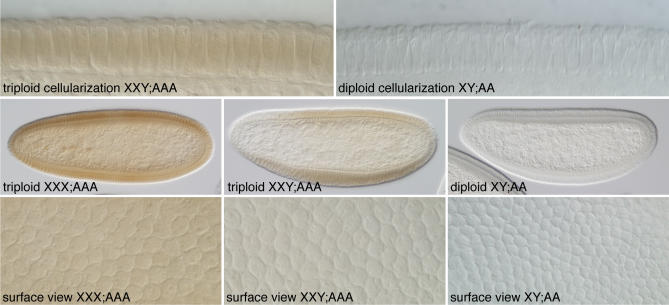
Triploid Embryos Cellularize in Nuclear Cycle 13 Embryos were immunostained for *Sxl* protein. (Top row) Cell membrane formation in a cycle 13 triploid embryo (presumed XXY;AAA) and in a cycle 14 diploid male (XY;AA). (Middle row) During cellularization, whole-embryo views reveal uniform SXL staining in presumed XXX;AAA triploid female, nonuniform SXL staining in presumed XXY;AAA triploid intersex, and unstained diploid male. (Bottom row) Magnified surface views of same embryos showing nuclear densities during cellularization. Embryos were progeny of *gyn-2; gyn-3* females and *gyn-2; gyn-3* males.

We first examined cellularizing embryos from *gyn-2; gyn-3* mothers for nuclear and cell morphology and for the presence of *Sxl* protein. As expected, most embryos were indistinguishable from normal diploid females and males. They cellularized at cycle 14 nuclear density and either expressed SXL uniformly or not at all [43]. A small proportion of embryos from *gyn-2; gyn-3* mothers; however, displayed unusual phenotypes. These rare progeny possessed relatively large nuclei and cellularized at cycle 13 nuclear density (Figure 4). These prematurely cellularizing embryos could be subdivided based on their pattern of *Sxl* protein staining. Half stained strongly and uniformly for SXL, whereas the other half exhibited weaker, nonuniform SXL staining, suggesting that they represented XXX;AAA and XXY;AAA embryos, respectively (Figure 4). Taken at face value, these data imply that triploids cellularize during nuclear cycle 13. This suggests that XX;AAA triploids may be sexual mosaics, not because of their intermediate X:A ratio, but rather because premature cessation of the X-counting process produces too low levels of XSE products to reliably activate *SxlPe* during the abbreviated syncytial blastoderm stage. Triploid XXX;AAA embryos would by this logic be female, because the three X chromosomes would supply sufficient XSE proteins to strongly activate *SxlPe* and the three copies of *Sxl* would produce enough SXL to reliably engage autoregulation.

### Expression from *SxlPe* in Triploid Embryos

**Figure 5:**
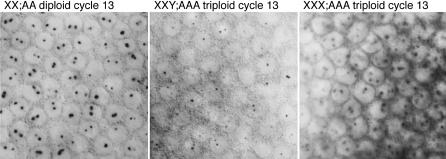
Nascent *SxlPe*-Derived Transcripts in Diploid and Triploid Embryos Surface views of cycle 13 embryos. Diploids cellularize during cycle 14 and triploids during cycle 13. Embryos were progeny of *gyn-2; gyn-3* females and *gyn-2; gyn-3* males.

**Figure 6:**
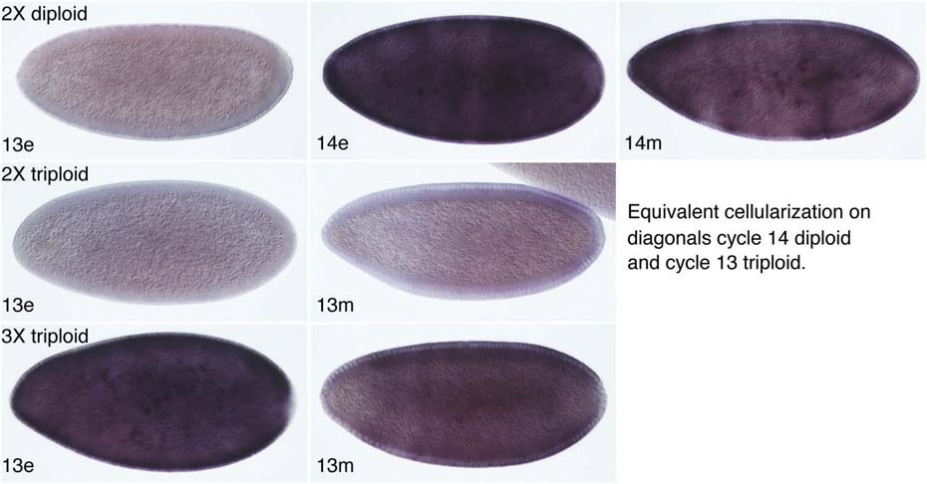
Accumulation of *SxlPe* mRNA in Triploid and Diploid Embryos Diploid female (top row), triploid XXY;AAA (middle row), and triploid XXX;AAA (bottom row) embryos were stained after in situ hybridization to detect *SxlPe*-derived transcripts. Nuclear cycles 13 and 14 are marked: e and m denote estimated early (≤5 min) and mid (≤30 min) stages of indicated cycles. Embryos were progeny of *gyn-2; gyn-3* females and *gyn-2; gyn-3* males. Orientation is anterior to the left and dorsal to the top.

To confirm that triploid embryos cellularize at cycle 13 nuclear density and to monitor the effect of premature cellularization on *SxlPe*, we examined embryos from *gyn2; gyn3* mothers using in situ hybridization. Triploid XXX;AAA females were expected to display three nuclear dots indicating *Sxl* transcription from all three X chromosomes. We observed embryos with three nuclear dots and cycle 12 or 13 nuclear densities, but found none that had three dots and cycle 14 nuclear density. Many of those with three nuclear dots and cycle 13 density had begun to cellularize, confirming that triploid embryos undergo cellularization during nuclear cycle 13 (Figures 5 and 6). Examination embryos with two nuclear dots revealed they were of two kinds: normal diploid females with uniform *SxlPe* expression in cycle 13 and high levels of cytoplasmic *Sxl* mRNA in cycle 14, and the presumed XXY;AAA triploids, distinguishable by their weaker, nonuniform *SxlPe* expression and mRNA staining, and by their undergoing cellularization during cycle 13 (Figures 5 and 6).

Our findings with triploid embryos support the hypothesis that the flies determine their sex by measuring the concentration of XSE proteins in the precellular cycles, rather than by reading the value of the X:A ratio. The intersexuality of XX;AAA flies, traditionally attributed to a decrease in the ratio of female-determining to male-determining proteins, can be more accurately explained as an indirect effect of autosomal ploidy on the timing of embryonic cell cycles. In this view, XX;AAA embryos mimic diploid females until early cycle 13. From that point on, however, premature cessation of the X-counting process leads to less-efficient expression from *SxlPe* and the failure to reliably engage *Sxl* autoregulation, creating embryos that are mosaics for *Sxl* expression. A related phenomenon has been described for mutations affecting JAK/STAT signaling during sex determination. In that case, failure to maintain high-level *SxlPe* expression during diploid cycle 14 led to reductions in autoregulated *Sxl* expression, generating sexually mosaic embryos analogous to those described here [29].

## Discussion

Normal *Drosophila* are males if their cells possess one X chromosome and females if they have two Xs. Demonstration of this fact was central to Calvin Bridges' 1916 proof that genes are located on chromosomes [44]. The contemporary notion that sex is signaled not by X number, but by the value of the X:A ratio, stems from Bridges' work [3,4] showing that possession of two Xs was not sufficient to determine the female fate in triploid flies. Despite the long-standing acceptance of the X:A hypothesis, the evidence that the value of the X:A ratio determines sex is largely correlative and indirect. Fundamentally, the X:A model rests on correlations between adult sexual phenotypes and the value of the ratio in several karyotypes (Table 1). However, the inference that the value of the X:A ratio is instructive, and not merely predictive, as to sex, depends on the assumption that normal adult sexual phenotypes reflect normal operation of earlier regulatory events (see [1,14]). Our demonstration that changes in ploidy alter the temporal and developmental contexts in which sex is assessed shows this underlying assumption to be flawed. Haploidy alters sex determination by increasing the time when the sex signal is assessed; triploidy acts reciprocally, by compressing the time available. Both conditions alter the response of *SxlPe* to the sex signal in ways that suggest the promoter responds primarily to the concentrations of XSE products present in the embryo rather than to the particular value of the X:A ratio.

This revised view of sex determination is not entirely new. In 1934, Dobzhansky and Schultz [45] offered a cautionary alternative to the X:A hypothesis, warning that the influence of the autosomes on sex may be indirect. Their proposal, that “sex may be determined by the ratio between the number of X chromosomes present in the cell and the size of that cell” differs in specifics from our findings, but the fundamental logic is the same. In 1983, Baker and Belote [46] suggested that maternally contributed products, rather than autosomal factors, represented the key reference to which X dose is measured. More recently, Cline and colleagues [15–18], have pointed out the logical similarity of autosomal elements to maternal elements and highlighted the weak quantitative role of *dpn*, the sole autosomal element [17,18]. Our findings extend these critical evaluations of the X:A hypothesis, providing a mechanistic explanation for why the ratio may predict sex without specifying it, and offering experimental support for the idea that the primary sex-determination signal is better described as the dose of XSE genes than as the X:A ratio.

It could be argued that the differences between XSE-sensing and X:A-reading models are largely semantic. Both predict sex and both accommodate the same set of XSEs, maternal factors, and autosomal repressor. This, however, is more than an argument about the meaning of words. The XSE-sensing model has the advantages of clarifying terminology, erasing artificial distinctions between maternal and zygotic elements of the sex-signaling system, and providing a more concrete and accurate concept of mechanism. In the conventional X:A paradigm, autosomal “denominator elements” are necessarily core components of the X:A signal, whereas maternal factors are consigned to “X:A signal-transducing” roles [1,14]. This logical formalism creates a situation in which the sole denominator element, the relatively weak *dpn*, is a more central part of the sex signal than the more numerous and potent maternal signal-transducing factors including, Daughterless, the dimerization partner of the XSE protein Scute, Stat92E, the transcription factor signaled to bind *SxlPe* by the XSE *unpaired*, and Groucho, the corepressor needed for Dpn function at *SxlPe*. XSE-sensing mechanisms avoid such confusion by treating the maternal and autosomal signal element (MSE and ASE) proteins as parts of the cellular and biochemical context in which male and female XSE concentrations are assessed [1,15–18,37]. Reclassification of autosomal factors from X:A denominator elements to “context genes” [1] also serves to highlight the importance of the dynamic temporal and cellular milieu in which X chromosome counting occurs. Nuclear cycles 8–14 represent the transition from maternal to zygotic control of gene expression [47]. The timing of XSE gene and *SxlPe* activation, suggests that sex determination is directly connected to more-general events occurring at the mid-blastula transition. Factors such as changing chromatin environments and the timed onset of general zygotic gene expression mediated by Bicoid stability factor (BSF) [28,47], or other timing factors, seem likely to influence *SxlPe*'s threshold response. Perhaps equally important, global events associated with the mid-blastula transition may couple the inactivation of XSE transcription and the rapid degradation of XSE and MSE mRNAs to the onset of cellularization [47,48]. If these events are responsible for the timely shut down of *SxlPe* (see [16,49]), they further highlight the role developmental context plays in preventing XY;AA diploids from activating *SxlPe* during cycle 14, and in explaining the sexual mosaicism of XXY;AAA animals.

In terms of transcriptional mechanisms, XSE-sensing schemes have the advantage of replacing the incorporeal concept of the value of the X:A ratio with the tangible notion that threshold concentrations of XSE proteins activate *SxlPe*. Models for how XSE thresholds are set need not invoke the conjectural titrations of XSE proteins by ASEs that seem inevitably to arise from the need to explain how the X:A ratio is read (see [5–7,49]). Instead, one can focus on how dose sensitivity might be explained by the known activators and repressors acting at *SxlPe*. The *Drosophila* dorsal–ventral and anterior–posterior patterning systems, in which enhancers integrate positive and negative inputs over narrow concentration ranges, provide precedents for how on or off decisions can be regulated by DNA-binding proteins (see [50–52]).

Although our findings, and those of others, suggest a more realistic approach to mechanism, our data on the correlations between XSE expression and timing of *SxlPe* activation raise something of a paradox. The modern form of Dobzhansky and Shultz's 1934 argument [45], that the changes in nuclear volume that accompany changes in ploidy might account for the predictive effects of the X:A ratio [15,45], would suggest that, for any given stage, the XSE concentrations in small 1X haploid nuclei should be similar to those found in larger 2X diploid nuclei, and thus, that *Sxl* expression should occur with similar timing in haploids and diploids. Given the absence of information on XSE protein concentrations, the apparent conflict between our observations and expectations based on nuclear volume is currently unresolvable; however, it cautions that factors in addition to relative XSE gene expression may influence the timing of *SxlPe* activation. Regardless, either view supports the argument that it is inappropriate to consider the value of the X:A ratio as a simple sex-determining signal [15,17,18,45,46]. Rather, both suggest that the sex-determination signal should be defined in the normal diploid context, in which differential X chromosome dose specifies sex by determining the concentrations of XSE products present in the embryo.

Looking beyond *Drosophila*, our reinterpretation of the effects of ploidy on primary sex determination has implications for other developmental systems that rely on differential doses of chromosomes to define sexual fates. These include systems thought to read X:A ratios, and one, that as traditionally viewed, cannot.

Haplodiploidy is a widespread means of sex determination in which haploids develop as males and diploids as females. The best understood example of haplodiploidy is complementary sex determination (CSD), known to occur in many bee and wasp species [53–55]. In CSD, females are heterozygous, and males hemizygous, for one sex-determining locus with multiple alleles. Although the CSD mechanism is unrelated to the X-counting process of the fruit fly [56], many haplodiploid species and genera lack CSD. The traditional X:A model of *Drosophila* is difficult to reconcile with haplodiploidy because the X:A balance is the same regardless of ploidy [57]. Our findings, however, suggest that a *Drosophila*-like chromosome-counting mechanism could operate in non-CSD haplodiploid species, if haploid and diploid zygotic gene doses were measured in similar cellular contexts. Presciently, Crozier [54,58] proposed that a variation of the *Drosophila* mechanism based on the chromosomal/cytoplasmic balance could distinguish haploid and diploid embryos. Such a chromosome-measuring system would have a strong maternal component [55,58] that could exhibit strain or species variation consistent with extensive involvement of the maternal genome in many insect sex-determining systems [53].

Mammalian sex depends on the Y chromosome, but a second aspect of sexual dimorphism, X chromosome inactivation, requires that X dose be assessed. It is thought that X counting in mammalian cells depends on the X:A ratio, because the number of active Xs increases with the number of autosome sets (see [2,59]). Recent models of the establishment of X inactivation incorporate the X:A concept; invoking titrations of autosome-encoded factors by X-linked sites [59–61] that bear remarkable similarity to early speculations as to how the fly X:A ratio might be read (see [46]). However, new findings suggesting a role for X chromosome pairing in X counting and choice [62,63], and indications that ploidy has less impact on X inactivation than generally thought [64] are difficult to reconcile with traditional notions of the X:A ratio. In this light, our findings caution that abnormal ploidy may also alter the cellular context in which mammalian X counting occurs. If so, the effects of altered ploidy may suggest only that autosomal products function in the X-counting process and not that they are a central part of a specific X:A signaling mechanism.

Of the well-known experimental systems said to depend on X:A ratios, it may be that only the nematode C. elegans actively consults the X:A balance when measuring X chromosome dose [65]. Why might assessment of the X:A ratio be central to worm sex, but only a minor aspect of the fruit fly mechanism? Perhaps the structures of the regulatory systems dictated their evolution. Superficially, the C. elegans mechanism resembles that of the fruit fly in that at least four XSE gene products regulate the expression state of a single sex-determining switch gene, *xol-1* [66,67]. However, in C. elegans, the XSEs antagonize the actions of several discrete ASEs that function to activate *xol-1* in males [65]; whereas in *Drosophila*, the XSEs activate their target, *Sxl*. For the fly, it is possible to envision how an ancestral X chromosome–counting mechanism, based on XSE dose and maternal factors, could have differentially expressed *Sxl*, and how the autosomal element, *dpn*, could later have been added to refine the regulation of *Sxl* [68]. In contrast, for C. elegans, autosomal elements must have been involved from the beginning, for without ASE-mediated activation of *xol-1*, the repressive sex-determining functions of the XSEs would have been moot. Whether C. elegans primary sex determination also relies on an extensive maternal contribution remains to be determined.

## Materials and Methods

### Generation of haploid, diploid, and triploid embryos.

Haploid embryos were from females homozygous for the recessive X-linked maternal-effect mutations, *maternal haploid* (*mh^1^*) [30,35] or *sesame* (*ssm^185b^*), also known as *Hira* [33]. Diploid control embryos from sibling females heterozygous for *mh* (*z^1^ w mh^1^/FM3* X *z^1^ w mh^1^*/Y) or *ssm* (*w ssm^185b^*/FM7 X *w ssm^185b^/*Y) were indistinguishable from embryos from wildtype stocks. Eggs from *mh^1^/mh^1^* and *ssm^185b^/ssm^185b^* females develop as maternally derived (gynogenetic) haploids because, for *mh^1^*, the paternally derived sister chromatids fail to separate during the first embryonic mitosis, leading to their loss during the next three divisions [34], or for *ssm^185b^*, because the male pronucleus does not fully decondense, is arrested before the first S-phase, and fails to enter the first mitotic spindle [35]. Haploid embryos from *mh^1^* or *ssm^185b^* mothers were indistinguishable with respect to *Sxl* and XSE gene expression (unpublished data). Our initial analysis of haploids used *mh^1^*, but most later experiments exploited *ssm^185b^* because we found that a fraction of embryos derived from *mh^1^*, but not *ssm^185b^*, mothers were partial diploids and that others appeared to have lost the X chromosome in some of their nuclei (unpublished data). Triploid embryos and sibling diploid controls were generated from mothers homozygous for two recessive maternal effect mutations *gynogenetic-2* and *-3* (*gyn-2* and *gyn-3*) [40]. Most eggs laid by homozygous *gyn-2; gyn-3* females are haploid and develop as normal diploid embryos when fertilized; however, *gyn-2;gyn-3* mothers produce a small and variable percentage of diploid eggs that develop as XXX;AAA or XXY;AAA triploids depending on whether they are fertilized by an X-bearing or Y-bearing sperm [40,41]. The *Drosophila* Y chromosome does not influence sex determination. Stock *z^1^ w mh^1^*/*FM3* was provided by M. Wolfner (Cornell University), *w ssm^185b^*/*FM7* was from B. Loppin (Centre de Génétique Moléculaire et Cellulaire), and *w^1^; gyn-2; gyn-3* was from the Bloomington *Drosophila* Stock Center.

### In situ hybridization, immunostaining, and embryo staging.

Embryos were collected, and processed for immunocytochemistry according to Patel et al. [66]. Anti-Sxl mouse antibody (gift of T. Cline, University of California, Berkeley) was used at 1:300 dilution. Horseradish peroxidase secondary antibodies (Jackson ImmunoResearch) were used at a dilution of 1:300 and visualized with 3,3′diaminobenzidine. All embryos were stained with DAPI to visualize DNA. In situ hybridization was done using standard procedures as described [16,17,27,29,36,70]. Briefly, digoxygenin-labeled RNA probes complementary to *Sxl* exon E1 or the *scute* coding regions [16] were prepared using in vitro transcription of plasmid or PCR-derived templates. *Sxl* exon E1 probes detect both *SxlPe*-derived mRNA and *Pe*-derived nascent transcripts, the latter visible as focused dots of staining within nuclei. *Sxl* and *scute* are X-linked so the number of nuclear dots corresponds to the number of X chromosomes. In all cases, we analyzed expression of the endogenous loci. No transgenic promoter fusions were used. Because haploid embryos and their diploid controls derived from different females, embryos were collected, processed, and hybridized in parallel. Triploid embryos and their diploid control siblings were from the same egg collections. For embryo staging, cell cycle number was determined by nuclear density [30,32,71]. Nuclei change in size and appearance as they progress through the precellularization cycles [72], and we exploited this to stage embryos as closely as possible. Timing through the cellularization cycles was estimated by nuclear shape and length, by the distance from the base of the nucleus to the yolk, and by the extent of membrane furrow invagination [32,71]. Detailed comparisons of cell cycles and gene expression in haploid and diploid embryos have been published [30–32]. Time estimates for the cellularization cycle in triploids was by analogy to diploid and haploid embryos. An abundant literature (see [30,32]) has established that the timing of the mid-blastula transition is linked to the ratio of DNA to cytoplasm in the embryos of many species. Cleavage divisions stop and cellularization cycles begin when the nucleocytoplasmic ratio reaches a threshold value, explaining why haploids undergo one more cleavage division and tetraploids one fewer division than diploid embryos [32].

### Numbers of embryos examined.

The figures summarize the results of many different experiments with haploid, diploid, and triploid embryos. Only some experiments were quantified by counting the number of embryos at specific stages, but the results were qualitatively assessed as the same for each repetition. The following represent numbers of embryos counted and recorded with respect to the listed conclusions, but many others were observed. Timing of *SxlPe* activation in haploids: 10 cycle: 11 embryos, 28 cycle: 12 embryos, 32 cycle: 13 embryos, 51 cycle: 14 embryos, and 42 cycle: 15 embryos. Timing of *SxlPe* activation in diploids has been established [16,17,27,29], but we note that about one fourth (11 of 39) of wild-type cycle 12 embryos exhibited detectable *Sxl* expression, consistent with the onset occurring in females during cycle 12. The time course of *scute* expression in Figure 3 was assembled from photographs of every cycle 12, 13, and 14 haploid embryo, every cycle 12 and 13 diploid embryo, and from 13 haploid and 10 diploid embryos in the cellularization cycles in the experiment. The embryos shown were judged as close in stage as possible based on the density, size, and morphology of DAPI-stained nuclei [32,72]. The percentage of triploids among diploid progeny of *gyn-2, gyn-3* mothers was variable [40] for unknown reasons. The fraction of pre-germ band–extended triploids with mosaic SXL staining (presumed XXY;AAA) was about 50% in all experiments (21/39 counted). We counted 17 XXX;AAA and 14 presumed XXY;AAA embryos that expressed *SxlPe*, but observed numerous others.

## Abbreviations

ASE: autosomal signal element
CSD: complementary sex determination
X:A ratio: ratio of X chromosomes to autosomal sets
XSE: X-linked signal element

## References

[pbio-0050332-b001] Cline TW, Meyer BJ (1996). Vive la difference: males vs females in flies vs worms. Annu Rev Genet.

[pbio-0050332-b002] Avner P, Heard E (2001). X-chromosome inactivation: counting, choice and initiation. Nat Rev Genet.

[pbio-0050332-b003] Bridges CB (1921). Triploid intersexes in Drosophila melanogaster. Science.

[pbio-0050332-b004] Bridges CB (1925). Sex in relation to chromosomes and genes. Am Nat.

[pbio-0050332-b005] Schutt C, Nothiger R (2000). Structure, function and evolution of sex-determining systems in Dipteran insects. Development.

[pbio-0050332-b006] Hartwell L, Hood L, Goldberg M, Reynolds A, Silver L (2004). Genetics: from genes to genomes. 2nd edition.

[pbio-0050332-b007] Gilbert SF (2006). Developmental biology. 8th edition.

[pbio-0050332-b008] Keyes LN, Cline TW P. S (1992). The primary sex determination signal of Drosophila acts at the level of transcription. Cell.

[pbio-0050332-b009] Bell LR, Horabin JI, Schedl P, Cline TW (1991). Positive autoregulation of Sex-lethal by alternative splicing maintains the female determined state in Drosophila. Cell.

[pbio-0050332-b010] Nagengast AA, Stitzinger SM, Tseng CH, Mount SM, Salz HK (2003). Sex-lethal splicing autoregulation in vivo: interactions between SEX-LETHAL, the U1 snRNP and U2AF underlie male exon skipping. Development.

[pbio-0050332-b011] Christiansen AE, Keisman EL, Ahmad SM, Baker BS (2002). Sex comes in from the cold: the integration of sex and pattern. Trends Genet.

[pbio-0050332-b012] Ashburner M, Golic KG, Hawley RS (2005). Drosophila: a laboratory handbook.

[pbio-0050332-b013] Lucchesi JC, Kelly WG, Panning B (2005). Chromatin remodeling in dosage compensation. Ann Rev Genet.

[pbio-0050332-b014] Cline TW (1988). Evidence that sisterless-a and sisterless-b are two of several discrete “numerator elements” of the X:A sex determination signal in Drosophila that switch Sex-lethal between two alternative stable expression states. Genetics.

[pbio-0050332-b015] Cline TW (1993). The Drosophila sex determination signal: how do flies count to two?. Trends Gen.

[pbio-0050332-b016] Erickson JW, Cline TW (1993). A bZIP protein, SISTERLESS-A, collaborates with bHLH transcription factors early in Drosophila development to determine sex. Genes Dev.

[pbio-0050332-b017] Barbash DA, Cline TW (1995). Genetic and molecular analysis of the autosomal component of the primary sex determination signal of Drosophila melanogaster. Genetics.

[pbio-0050332-b018] Wrischnik LA, Timmer JR, Megna LA, Cline TW (2003). Recruitment of the proneural gene scute to the Drosophila sex-determination pathway. Genetics.

[pbio-0050332-b019] Younger-Shepherd S, Vaessin H, Bier E, Jan LY, Jan YN (1992). deadpan, an essential pan-neural gene encoding an HLH protein, acts as a denominator element in Drosophila sex determination. Cell.

[pbio-0050332-b020] Bridges C (1930). Haploid Drosophila and the theory of genic balance. Science.

[pbio-0050332-b021] Crew FAE, Lamy R (1938). Fertile haploid sectors by partial merogony in mozaics of Drosophila pseudo-obscura. Nature.

[pbio-0050332-b022] Waletzky E (1937). A haploid mosaic of Drosophila melanogaster. Drosoph Info Ser.

[pbio-0050332-b023] Santamaria P (1983). Analysis of haploid mosaics in Drosophila. Dev Biol.

[pbio-0050332-b024] Fuyama Y (1987). Haploid embryos of Drosophila melanogaster are female. Drosoph Info Serv.

[pbio-0050332-b025] Sanchez L, Nothiger R (1983). Sex determination and dosage compensation in Drosophila melanogaster: production of male clones in XX females. EMBO J.

[pbio-0050332-b026] Cline TW (1984). Autoregulatory functioning of a Drosophila gene product that establishes and maintains the sexually determined state. Genetics.

[pbio-0050332-b027] Erickson JW, Cline TW (1998). Key aspects of the primary sex determination mechanism are conserved across the genus Drosophila. Development.

[pbio-0050332-b028] Bosch JR, Benavides JA, Cline TW (2006). The TAGteam DNA motif controls the timing of Drosophila pre-blastoderm transcription. Development.

[pbio-0050332-b029] Avila FW, Erickson JW (2007). Drosophila JAK/STAT pathway reveals distinct initiation and reinforcement steps in early transcription of Sxl. Curr Biol.

[pbio-0050332-b030] Edgar BA, Kiehle CP, Schubiger G (1986). Cell cycle control by the nucleo-cytoplasmic ratio in early Drosophila development. Cell.

[pbio-0050332-b031] Rose LS, Wieschaus E (1992). The Drosophila cellularization gene nullo produces a blastoderm-specific transcript whose levels respond to the nucleocytoplasmic ratio. Genes Dev.

[pbio-0050332-b032] Grosshans J, Muller HA, Wieschaus E (2003). Control of cleavage cycles in Drosophila embryos by fruhstart. Dev Cell.

[pbio-0050332-b033] Loppin B, Docquier M, Bonneton F, Couble P (2000). The maternal effect mutation sesame affects the formation of the male pronucleus in Drosophila melanogaster. Dev Biol.

[pbio-0050332-b034] Loppin B, Berger F, Couble P (2001). The Drosophila maternal gene sesame is required for sperm chromatin remodeling at fertilization. Chromosoma.

[pbio-0050332-b035] Loppin B, Berger F, Couble P (2001). Paternal chromosome incorporation into the zygote nucleus is controlled by maternal haploid in Drosophila. Dev Biol.

[pbio-0050332-b036] Shermoen AW, O'Farrell PH (1991). Progression of the cell cycle through mitosis leads to the abortion of nascent transcripts. Cell.

[pbio-0050332-b037] Yang D, Lu H, Hong Y, Jinks TM, Estes PA (2001). Interpretation of X chromosome dose at Sex-lethal requires non-E-box sites for the basic helix-loop-helix proteins SISB and daughterless. Mol Cell Biol.

[pbio-0050332-b038] Torres M, Sanchez L (1991). The sisterless-b function of the Drosophila gene scute is restricted to the stage when the X:A ratio determines the activity of Sex-lethal. Development.

[pbio-0050332-b039] Deshpande G, Stukey J, Schedl P (1995). scute (sis-b) function in Drosophila sex determination. Mol Cell Biol.

[pbio-0050332-b040] Fuyama Y (1986). Genetics of parthenogenesis in Drosophila melanogaster. II. Characterization of a gynogenetically reproducing strain. Genetics.

[pbio-0050332-b041] Presgraves DC (2000). A genetic test of the mechanism of Wolbachia-induced cytoplasmic incompatability in Drosophila. Genetics.

[pbio-0050332-b042] Cline TW (1983). The interaction between daughterless and Sex-lethal in triploids: a lethal sex-transforming maternal effect linking sex determination and dosage compensation in Drosophila melanogaster. Dev Biol.

[pbio-0050332-b043] Bopp D, Bell LR, Cline TW, Schedl P (1991). Developmental distribution of female specific Sex-lethal proteins in Drosophila. Genes Dev.

[pbio-0050332-b044] Bridges CB (1916). Non-disjunction as proof of the chromosome theory of heredity. Genetics.

[pbio-0050332-b045] Dobzhansky T, Schultz J (1934). The distribution of sex-factors in the X-chromosome of Drosophila melanogaster. J Genet.

[pbio-0050332-b046] Baker BS, Belote JM (1983). Sex determination and dosage compensation in Drosophila melanogaster. Ann Rev Genet.

[pbio-0050332-b047] De Renzis S, Elemento O, Tavazoie S, Wieschaus EF (2007). Unmasking activation of the zygotic genome using chromosomal deletions in the Drosophila embryo. PLoS Biol.

[pbio-0050332-b048] Bashirullah A, Cooperstock RL, Lipshitz HD (2001). Spatial and temporal control of RNA stability. Proc Natl Acad Sci U S A.

[pbio-0050332-b049] Louis M, Holm L, Sanchez L, Kaufman M (2003). A theoretical model for the regulation of Sex-lethal, a gene that controls sex determination and dosage compensation in Drosophila melanogaster. Genetics.

[pbio-0050332-b050] Ochoa-Espinosa A, Yucel G, Kaplan L, Pare A, Pura N (2005). The role of binding site cluster strength in Bicoid-dependent patterning in Drosophila. Proc Natl Acad Sci U S A.

[pbio-0050332-b051] Zinzen RP, Senger K, Levine M, Papatsenko D (2006). Computational models for neurogenic gene expression in the Drosophila embryo. Curr Biol.

[pbio-0050332-b052] Arnosti DN (2002). Design and function of transcriptional switches in Drosophila. Insect Biochem Mol Biol.

[pbio-0050332-b053] Normark BB (2003). The evolution of alternative genetic systems in insects. Annu Rev Entomol.

[pbio-0050332-b054] Crozier R (1971). Heterozygosity and sex determination in haplodiploidy. Am Nat.

[pbio-0050332-b055] Cook J (1993). Sex determination in the Hymenoptera: a review of models and evidence. Heredity.

[pbio-0050332-b056] Beye M, Hasselmann M, Fondrk MK, Page RE, Omholt SW (2003). The gene csd is the primary signal for sexual development in the honeybee and encodes an SR-type protein. Cell.

[pbio-0050332-b057] Charlesworth B (2003). Sex determination in the honeybee. Cell.

[pbio-0050332-b058] Crozier R (1977). Evolutionary genetics of the Hymenoptera. Annu Rev Entomol.

[pbio-0050332-b059] Plath K, Mlynarczyk-Evans S, Nusinow DA, Panning B (2002). Xist RNA and the mechanism of X chromosome inactivation. Annu Rev Genet.

[pbio-0050332-b060] Lee JT (2005). Regulation of X-chromosome counting by Tsix and Xite sequences. Science.

[pbio-0050332-b061] Whitelaw E (2006). Unravelling the X in sex. Dev Cell.

[pbio-0050332-b062] Xu N, Tsai CL, Lee JT (2006). Transient homologous chromosome pairing marks the onset of X inactivation. Science.

[pbio-0050332-b063] Patel NH, Goldstein LSB, Fyrberg EA (1994). Imaging neuronal subsets and other cell types in whole-mount drosophila embryos and larvae using antibody probes. Methods in cell biology.

[pbio-0050332-b064] Bacher CP, Guggiari M, Brors B, Augui S, Clerc P (2006). Transient colocalization of X-inactivation centres accompanies the initiation of X inactivation. Nat Cell Biol.

[pbio-0050332-b065] Gartler SM, Varadarajan KR, Luo P, Norwood TH, Canfield TK (2006). Abnormal X: autosome ratio, but normal X chromosome inactivation in human triploid cultures. BMC Genet.

[pbio-0050332-b066] Powell JR, Jow MM, Meyer BJ (2005). The T-box transcription factor SEA-1 is an autosomal element of the X:A signal that determines C. elegans sex. Dev Cell.

[pbio-0050332-b067] Nicoll M, Akerib C, Meyer BJ (1997). X-chromosome counting mechanisms that determine nematode sex. Nature.

[pbio-0050332-b068] Carmi I, Kopczynski JB, Meyer BJ (1998). The nuclear hormone receptor SEX-1 is an X-chromosome signal that determines nematode sex. Nature.

[pbio-0050332-b069] Pomiankowski A, Nothiger R, Wilkins A (2004). The evolution of the Drosophila sex-determination pathway. Genetics.

[pbio-0050332-b070] Lehmann R, Tautz D, Goldstein LSB, Fyrberg EA (1994). In situ hybridization to RNA. Drosophila melanogaster: practical uses in cell and molecular biology.

[pbio-0050332-b071] Foe VA, Odell GM, Edgar BA, Bate M, Martinez-Arias A (1993). Mitosis and morphogenesis in the Drosophila embryo: point and counterpoint. The development of Drosophila melanogaster.

[pbio-0050332-b072] Edgar BA, Sprenger F, Duronio RJ, Leopold P, O'Farrell PH (1994). Distinct molecular mechanisms regulate cell cycle timing at successive stages of Drosophila embryogenesis. Genes Dev.

[pbio-0050332-b073] Novitski E (1984). Search for a tetraploid male. Drosoph Info Ser.

